# Where metabolism and virulence meet—sulfoxide reductases as determinants of bacterial survival

**DOI:** 10.1042/BST20250034

**Published:** 2026-08-17

**Authors:** Ulrike Kappler

**Affiliations:** School of Chemistry and Molecular Biosciences, The University of Queensland, St. Lucia, Qld 4072, Australia

**Keywords:** bacterial pathogens, energy generation, host-pathogen interactions, hypochlorite resistance, stress responses, Sulfoxide reduction

## Abstract

Bacterial metabolism is increasingly recognised as a driver of virulence in pathogens, and this includes the reduction of sulfoxides, which has traditionally been linked to anaerobic energy generation and maintenance of cellular proteins. Sulfoxide reduction is catalysed by two major enzyme groups, the Msr peptide methionine sulfoxide reductases that repair damaged proteins, and several types of mononuclear molybdenum enzymes that either convert small molecular S- and N-oxides or repair damaged proteins. Where these enzymes support virulence, they appear to be regulated by exposure to oxidising stressors such as hypochlorite and are often required for hypochlorite resistance and successful host interactions, either by maintaining the integrity of proteins or repairing damage to nutrient molecules such as amino acids and vitamins. As the virulence-supporting S- and N-oxide reductases retain their original architecture and links to metabolic processes, so that their action often also contributes to cellular redox balancing, energy generation, and access to specific nutrients, all of which support in-host survival.

## Introduction

Bacterial metabolism as a driver of niche adaptation is a key concept in environmental microbiology, and has emerged as an important factor that supports bacterial virulence and shapes the host–pathogen interface [1]. A common cellular reaction that has only recently been linked to bacterial virulence is the reduction of sulfoxides. Sulfoxides form naturally, for example as a result of degradation processes, as in the case of dimethylsulfoxide (DMSO), or when biological molecules such as sulfur-containing amino acids or vitamins are exposed to oxidising environments [2,3]. While the reduction of DMSO contributes to anaerobic energy generation, the reduction of sulfoxides (S-oxides) resulting from stress exposure is essential to maintain the biological function of the affected molecules and cellular survival [2,3].

**Figure 1:**
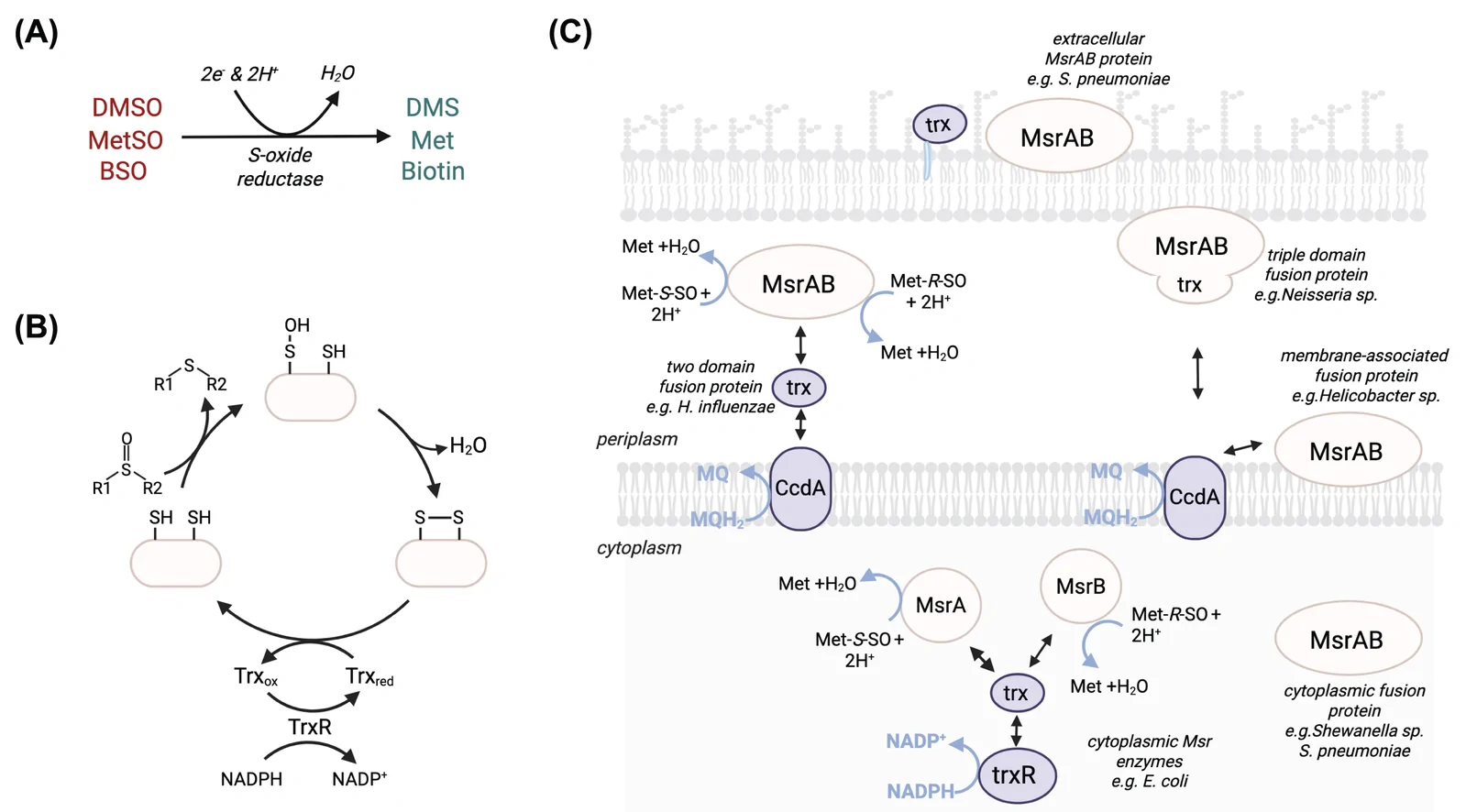
Sulfoxide reduction by Msr-type, thiol-based reductases (**A**) Schematic representation of sulfoxide reduction reactions. (**B**) Schematic representation of the catalytic mechanism of MsrA and MsrB thiol-based reductases, trx, thioredoxin; trxR, thioredoxin reductase; (**C**) Schematic representation of the structure and localization of thiol-based, Msr-type sulfoxide reductases. S_ox_, oxidized substrate; S_red_, reduced substrate; MQ/MQH_2_, menaquinone/ menaquinol; created using BioRender.com

In bacteria, two classes of enzymes that use fundamentally different catalytic mechanisms can mediate this reaction, the Msr Peptide Methionine Sulfoxide Reductases, and mononuclear molybdenum enzymes from two different enzyme families [2–4] (Figure 1A).

## Msr-type peptide methionine S-oxide reductases

Msr-type S-oxide reductases were first discovered in the 1980s, with the purification and characterisation of the cytoplasmic Methionine-*S*-sulfoxide (Met-*S*-SO)-specific MsrA enzyme [5]. The Met-*R*-SO-specific MsrB was only identified 20 years later [6], and belongs to a different enzyme family, even though the catalytic mechanisms of MsrA and MsrB are very similar (Figure 1B). Both MsrA and MsrB are able to repair oxidative damage to protein-bound methionine, as shown for the methionine-rich model protein, calmodulin [6], and several natural substrates that include bacterial adhesins [5]. Additionally, both enzymes have a limited ability to reduce free MetSO, where the activity of MsrA usually exceeds that of MsrB [6].

In addition to single-domain Msr proteins, a range of MsrAB fusion proteins has also been identified, predominantly in bacterial pathogens. In addition to simple two-domain fusion proteins such as MsrAB from *Haemophilus influenzae* [7], enzymes that contain additional domains, such as a thioredoxin domain in the *Neisseria* sp. MsrAB, membrane domains or lipoprotein signals have been characterized [8,9] (Figure 1C).

Catalysis in Msr-type MetSO reductases uses thiol-based chemistry that is generally mediated by two catalytic cysteine residues. The catalytic cysteine is required for a nucleophilic attack on the sulfur atom of MetSO, which, via a transition state, leads to the release of methionine and formation of a sulfenic acid group on the catalytic cysteine (Figure 1B). The second, so-called resolving cysteine, then reacts with the sulfenic acid group, forming a disulfide bond and releasing a water molecule. The two cysteines are re-reduced via reaction with thioredoxin, which in turn is reduced by a soluble or membrane-bound thioredoxin reductase (Figure 1B,C). While soluble thioredoxin reductases use NADPH as the electron donor, membrane-bound thioredoxin reductases use quinols as electron donors, thereby linking the enzymes to the bacterial respiratory chain.

## Mo-containing S-/N-oxide reductases

**Figure 2:**
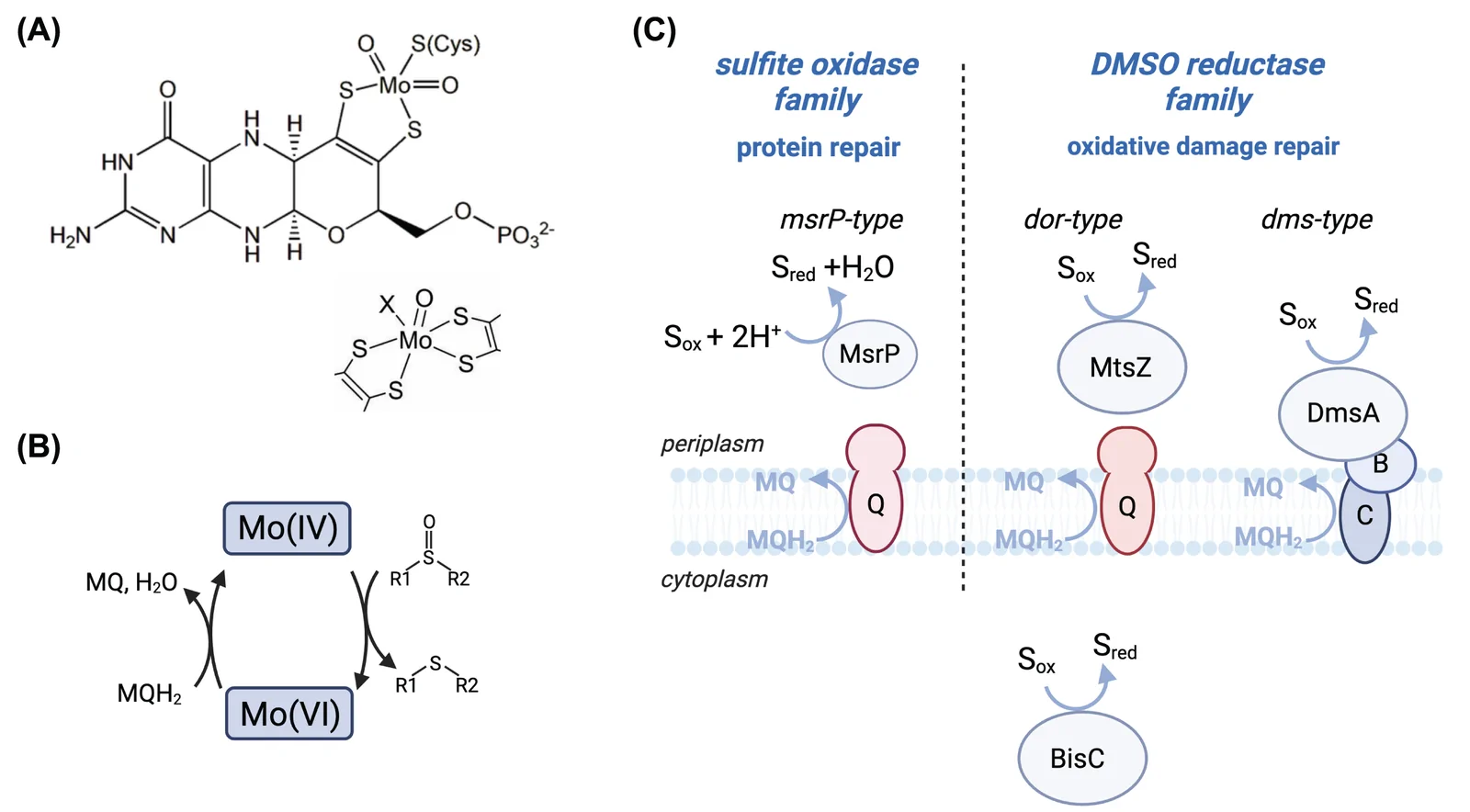
Sulfoxide reduction by Mo-containing reductases (**A**) Cofactors and Mo-centre coordination in Mo-containing sulfoxide reductases. Top—pyranopterin cofactor found in sulfite oxidase family enzymes such as MsrP; bottom—detail of Mo centre coordination in DMSO reductase family enzymes, showing the presence of two pyranopterin–dinucleotide cofactors. X-Mo ligand that varies between different types of enzymes. (**B**) Schematic representation of catalysis in Mo-containing sulfoxide reductases. (**C**) Schematic representation of the structure and localization of Mo-containing sulfoxide reductases. MsrP and BisC occur e.g. in *Escherichia coli*, MtsZ occurs e.g. in *H. influenzae*, DmsABC occurs e.g. in *E. coli*, *H. influenzae* and *Salmonella sp.* S_ox_, oxidized substrate; S_red_, reduced substrate; MQ/MQH_2_, menaquinone/menaquinol. Created using BioRender.com.

All Mo-containing S-oxide reductases contain one or two pyranopterin-type cofactors that complex the Mo atom which is the catalytic centre of these enzymes (Figure 2A,B). The best-studied Mo-containing S-oxide reductases are two structurally distinct enzymes: the three-subunit, membrane-bound DmsABC and the periplasmic DorA DMSO reductases, both of which belong to the DMSO reductase enzyme family [10,11]. Both enzymes also convert N-oxides and are connected to the bacterial quinone pool that is used as the electron donor via the integral membrane-anchor subunit, DmsC, and the membrane-bound DorC cytochrome, respectively.

Since their original discovery in *E.coli* and *Rhodobacter sp*., a range of enzymes related to DorA have been characterised in bacteria, including biotin and MetSO reductases [12–14]. In contrast, Dms-type enzymes have been mostly linked to DMSO reduction, but may also mediate iodate and bromate reduction as described in *Shewanella* species, and selenate reduction by the DmsA-related YnfE/YnfF reductases [3,15,16].

A third Mo-containing S-oxide reductase, MsrP (originally designated YedY) was more recently discovered and belongs to a different family of mononuclear Mo-enzymes, the sulfite oxidase family [17–19] (Figure 2C). The primary sequence of MsrP is unrelated to those of DmsA or DorA, however, similar to DorA, MsrP requires a membrane-bound cytochrome subunit, MsrQ, for electron transfer [18,19] (Figure 2C). MsrP was initially characterised as a weak DMSO reductase but has since been shown to reduce protein-bound MetSO, making its activity equivalent to that of MsrA and MsrB [17–19].

In Mo-containing S/N-oxide reductases, catalysis relies on the central Mo atom, where two electrons are transferred from the reduced Mo centre to the -oxo function of the sulfoxide group, forming an intermediate state where the sulfoxide is bound to the Mo centre [2,3]. The re-reduction of the now oxidised Mo centre eliminates the oxo-group via formation of water and involves electron-transfer subunits that use quinols as electron donors (Figure 2A,B).

Despite similarities in the basic catalytic mechanism, Mo-containing S/N-oxide reductases from the two enzyme families differ in the coordination and structure of the Mo centre (Figure 2A), as well as details of their catalytic mechanism, stereospecificity, and substrate preferences. The Dms- and Dor-type enzymes primarily reduce small-molecule S- and N-oxides such as DMSO and trimethylamine-N-oxide (TMAO) with *R*- and *S*-sulfoxide stereospecificity, respectively, while MsrP reduces both *R*- and *S*-sulfoxide forms of protein-bound MetSO residues [2,17,18].

## From cellular metabolism to virulence: functions of bacterial S-oxide reduction

The traditional roles of Msr- and Mo-containing S-oxide reductases are protein repair to maintain cellular integrity and anaerobic energy generation [2,3]. However, both types of enzymes have also been linked to bacterial survival in the host environment, which requires resistance to host-produced stressors, including diverse reactive oxygen (ROS) and chlorine species (RCS) such as hydrogen peroxide, superoxide, and hypochlorite [20]. Both ROS and RCS cause oxidative damage to cellular components and promote formation of S- and N-oxides on biological molecules [20].

For the thiol-based MsrA & MsrB enzymes, first links to virulence emerged with the discovery of their role in maintaining adhesins relevant for host–cell attachment in the late 1990s [5]. It is now well established that the activity of MsrA/B-type enzymes is required for resistance to hydrogen peroxide and hypochlorite in plant and mammalian pathogens such as *Salmonella enterica, Campylobacter jejuni, Mycoplasma, Enterococcus faecalis, H. influenzae* and *Xanthomonas* [7,21–25]. In all of these species, loss of either or both Msr enzymes also reduced virulence, presumably due to increased susceptibility to stress [7,21–25].

A particularly interesting aspect of this enzyme class is that despite their overall similar function, their cellular location can vary significantly. While most single-domain MsrA and MsrB enzymes are located in the bacterial cytoplasm, MsrA from *Streptococcus gordonii* is membrane-bound [26]. The MsrAB fusion proteins show the greatest diversity in cellular localisation [7–9,27–32] (Table 1). In *H. pylori*, MsrAB is a cell membrane-associated enzyme, while MsrAB from *H. influenzae* is a periplasmic enzyme that also associates with the outer membrane [7,28,29]. In *Neisseria* sp. MsrAB is an outer membrane-associated lipoprotein that contains a thioredoxin electron transfer domain and can face either the periplasm or the extracellular environment [8,9], while in *Streptococcus pneumoniae*, two fusion proteins exist, and MsrAB_2_ is a lipoprotein from *S. pneumoniae* that is located on the cell surface and interacts with specific lipo-protein thioredoxins that enable electron transfer [30] (Figure 2).

**Table 1:** MsrAB fusion proteins and their cellular locations

Bacteria	Domains	Redox partner	Location	Refs
*Neisseria sp.*	Trx-MsrAB	Fused Trx, DsbD	IC & OM, lipoprotein	[8]
*Haemophilus influenzae*	MsrAB	Trx, CcdA	Periplasm, (OM)	[7,31,33]
*Streptococcus pneumoniae*	MsrAB_1_; TM-MsrAB_2_	MsrAB_1_: Trx, TrxR MsrAB_2_: eTrx2, CcdA1,2	IC (MsrAB_1_) & CM (MsrAB_2_)	[30,34]
*Helicobacter pylori*	MsrAB	Trx1	CM & OMV;	[28,29,35]
*Fusobacterium nucleatum*	MsrAB; Trx-MsrAB^#^	Trx, CcdA CcdA	n.r.; may be exported*	[27]
*Treponema denticola*	MsrAB	n.r.	n.r,; exported*, pot. lipoprotein*	[36]
*Shewanella oneidensis*	MsrBA	Trx, TrxR	n.r.; IC*	[37]

**n.r.**, not reported; * predicted using SignalP 6.0 [38] **#** predicted using CDD database. In addition to the protein studied by Scheible et al [27], a three-domain fusion protein exists in *F. nucleatum* ATCC23726.

**Abbreviations:** IC, intracellular; OM, Outer Membrane; CM, Cytoplasmic Membrane, TM, Transmembrane domain; OMV, Outer Membrane Vesicles; Trx, thioredoxin; TrxR, Thioredoxin reductase; eTRX, extracellular thioredoxin with lipoprotein anchor.

In contrast, the link between Mo-based S-oxide reductases and virulence has only emerged in the past 10 years, beginning with the protein-repairing MsrP enzyme. MsrP was shown to be required for N-Chlorotaurine and chlorate resistance in bacteria such as *Salmonella Typhimurium* and *E. coli* [19,39,40], and repairs proteins required for cell envelope integrity such as the SurA chaperone and the Pal lipoprotein in both *E. coli* and *Salmonella* [19,39]. However, studies that explicitly test virulence-related phenotypes of *msrP* mutant strains are rare, and a recent study in *S. Typhimurium* demonstrated that MsrP contributes much less to oxidative stress resistance than MsrA or MsrB [41].

While the role of MsrP in promoting bacterial survival in the host is likely linked to its ability to repair damaged proteins, both Dor- and Dms-type sulfoxide reductases that only convert small molecule S-/N-oxides play significant roles in bacterial virulence. This was initially uncovered in strains of *H. influenzae*, which contain a single, conserved DmsABC S-oxide reductase and a *dor*-type MetSO reductase, MtsZ, that is present in 80% of strains [13,33,42]. The only natural niche of *H. influenzae*, the human respiratory tract, does not naturally contain DMSO or TMAO, which suggested a different physiological role than anaerobic energy generation. In keeping with a role in host–pathogen interactions, expression of both enzymes is induced by exposure to hypochlorite, and both were required for virulence in a mouse model of lung infection with DmsABC mutant strains showing stronger attenuation [13,33,42]. However, neither enzyme is required for resistance to hypochlorite. Possible natural substrates for DmsABC and MtsZ were identified as MetSO, nicotinamide-N-oxide, and pyrimidine-N-oxide, precursors for metabolites that *H. influenzae* is unable to synthesise, such as pyrimidine nucleobases and NAD [13,33,42]. In primary human nasal epithelia, *H. influenzae* strains lacking either *mtsZ* or *dmsA* showed reduced intracellular survival, but neither mutant strain had any appreciable phenotypes *in vitro* [13,33,42], demonstrating the close link between conditions encountered in the host and the requirement for these S-/N-oxide reductases, as well as an uncoupling of induction of enzyme expression by RCS from sensitivity to ROS/RCS.

Evidence from other studies supports these findings: DmsABC was also required for virulence of *Actinobacillus pleuropneumoniae* in pigs [43] and for colonisation of chickens by *S. Typhimurium*. However, this required inactivation of all three copies of *dmsA*-like genes present in *S. Typhimurium*, highlighting the significant redundancy of enzymes with S-oxide activity in *Salmonella* [44]. S-oxide reductase redundancy was also identified as a key factor in *E. coli* where hypochlorite-dependent expression of *dmsABC* modulated interactions with host cells, but the high redundancy of S-oxide reductases in this bacterium reduced the physiological effects of the mutation [45].

*Dor*-type S-/N-oxide reductases that are structurally related to *H. influenzae* MtsZ have been linked to host–pathogen interactions in other bacteria. In *S. Typhimurium*, the BisC biotin sulfoxide reductase was required for virulence, a phenotype linked to the availability of biotin [46]. Interestingly, in *E. coli* expression of several Mo-containing S-/N-oxide reductases was induced by exposure to oxidative stressors, suggesting that further studies of these enzymes and their role in host interactions are needed [45].

Another recent discovery is that different types of S-oxide reductases can form specific stress response systems. In *H. influenzae* three S-oxide reductases, MsrAB, MtsZ, and DmsABC, form a hypochlorite-inducible periplasmic stress response system where gene expression is controlled by the extracytoplasmic sigma factor RpoE2 [33,47]. In this stress response system, MsrAB is required for hypochlorite resistance mediated by repair of damaged periplasmic and cell envelope proteins, DmsABC and MtsZ mediate repair of oxidative damage to small substrate molecules [33] (Figure 2) that enhance nutrient availability during infection [7,31,33,42,47]. Both enzyme types also show direct links to host-interactions – MsrAB is involved in immunomodulatory processes that reduced expression of antimicrobial peptides during infection, while DmsABC and MtsZ were required for effective intracellular colonisation of host cells [31,33].

## Expression of virulence-associated S-oxide reductases is linked to exposure to ROS/RCS

Based on the available literature, a common feature of S- and N-oxide reductases that support bacterial virulence is that their expression is induced by different types of ROS or RCS and cell envelope damage rather than substrate molecules which is the case for enzymes such as the DorA DMSO reductase from *Rhodobacter sp*. that is required for growth of this bacterium with DMSO as an electron acceptor [33,45,48–50].

However, despite the common inducers, there is no apparent conservation of the regulators that control expression of S-oxide reductase genes in different bacterial species. ROS/RCS-dependent expression of *E. coli msrP* and *msrAB* from *F. nucleatum* is controlled by hypochlorite-responsive two-component regulatory systems [19,40,50], while the *msrAB* genes from *H. influenzae* and *Neisseria gonorrhoeae* and a putative *msrP* gene from *Azospira suillum* are controlled by extracytoplasmic function sigma factors [47,51,52]. Complex expression patterns have also been observed, e.g. in *Pseudomonas aeruginosa*, where *msrA* is constitutively expressed, while *msrB* expression is induced by hypochlorite [53]. Expression of virulence-associated S-oxide reductases thus appears to be independent of well-known oxidative stress regulators such as OxyR [25], and there is so far no evidence for a role of other characterised hypochlorite-responsive regulators [20]. This suggests that the regulatory mechanisms have evolved independently to suit the host environment and the specific physiology of each bacterial species.

## Do virulence associated S-oxide reductases still contribute to bacterial metabolism?

Despite the undisputable role of S-oxide reductases in supporting bacterial virulence, all Mo-containing S-/N-oxide reductases and some of the MsrAB fusion proteins are also connected to the bacterial respiratory chains as they use menaquinol as the electron donor (Figures 1C and 2C). As a result, the S-oxide reductases contribute to redox balancing and, depending on the respiratory chain configuration, also to energy generation. So, despite changes in how the enzymes are regulated and adaptations of substrate profiles for the Dms- and Dor-type enzymes to suit compounds available in the host, the S-oxide reductases still retain all functional aspects required for (anaerobic) energy generation, except that the electron acceptors may differ from those used by environmental bacteria, as the virulence-associated enzymes convert substrates available in host organisms. This dual functionality is particularly favourable for host-adapted pathogens such as *H. influenzae* that are in constant contact with host cells, i.e. S-oxide reductases are likely constantly expressed, which enhances nutrient availability and energy generation while at the same time reducing cellular stress levels and maintaining integrity of cellular components [33,45].

**Figure 3:**
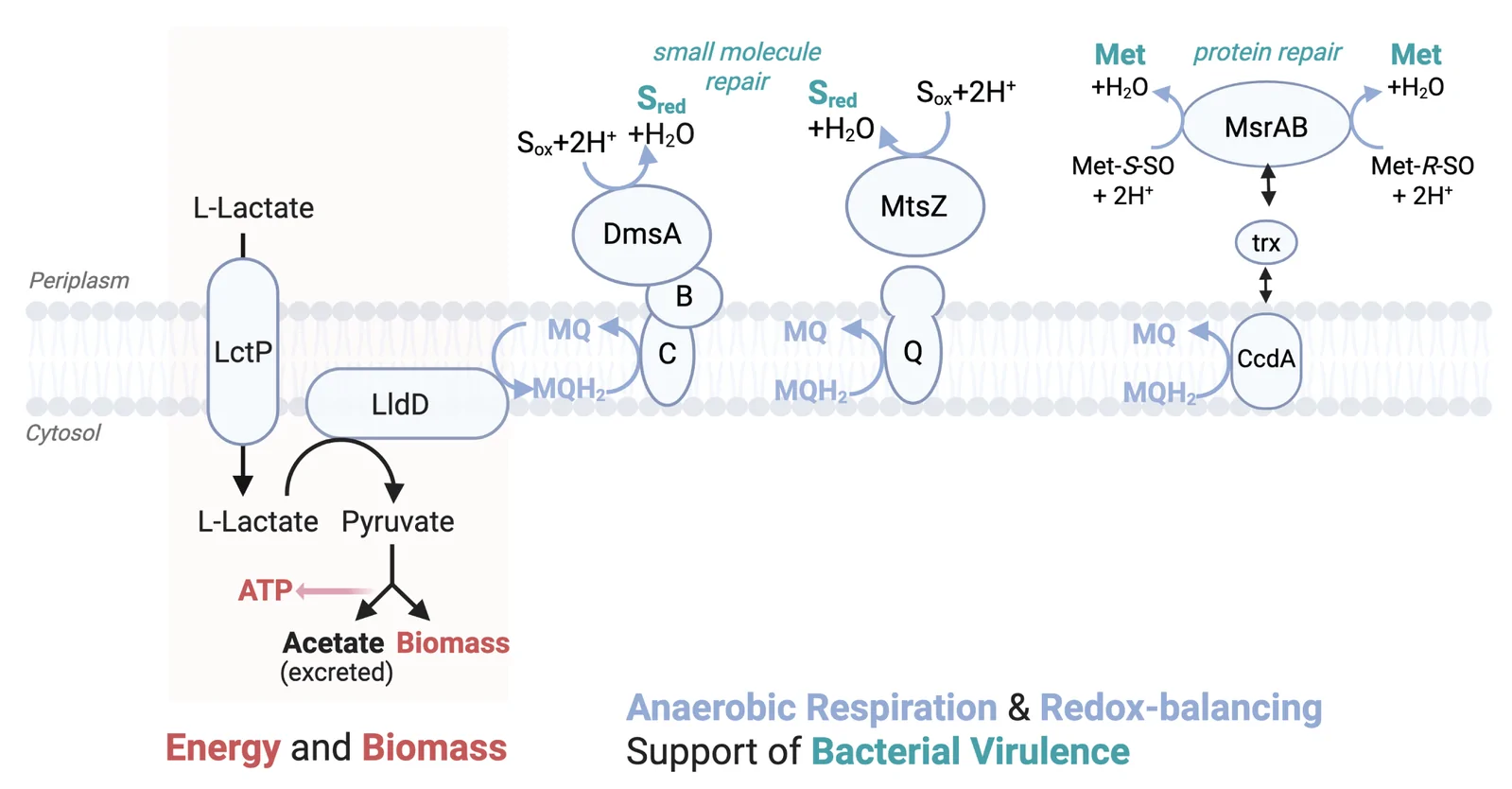
Connections between virulence supporting sulfoxide reductases, bacterial respiration and metabolism The enzymes shown occur in* H. influenzae*, but similar connections between respiration, metabolism, and virulence likely exist in many other bacterial species. Created using BioRender.com

However, virulence-associated S-oxide reductases can also provide other metabolic benefits to bacteria that exceed the obvious link to the respiratory chain and energy generation and can extend to enabling access to specific, difficult to metabolize growth substrates: In *H. influenzae*, the activity of S-oxide reductases during oxidative stress exposure can support the utilization of lactate, a key *H. influenzae* growth substrate during host contact. Growth on lactate requires respiratory processes to consume excess reducing equivalents produced during its utilisation as lactate is more reduced than cellular biomass [54], at the same time lactate is abundant during infection as host cells switch to anaerobic glycolysis. The main *H. influenzae* enzyme responsible for L-lactate utilization, LldD, reduces quinones to quinols, generating the electron donors for S-oxide reduction that would be switch on during infection as a result of exposure of *H. influenzae* to host-produced ROS/RCS (Figure 3). Similar processes may take place in other bacteria and connect metabolism and virulence, but require further exploration.

The dual roles of Msr-type and the Mo-containing S-oxide reductases in metabolism and virulence are likely a result of their evolutionarily ancient origins. These enzymes occur across all domains of life, and are also found in environmental bacteria that do not inhabit host organisms. In these bacteria, MsrA and MsrB maintain cellular integrity, while the Mo-containing S-oxide reductases mediate anaerobic energy generation with DMSO, as originally described [3,11]. Our recent analysis of DmsA phylogeny revealed a separation of sequences from bacterial pathogens versus environmental bacteria, as well as a wide distribution of homologous enzymes in other bacterial phyla, including in Gram-positive bacteria where DmsA is unstudied [45]. Similar diversity may exist for other S-oxide reductases. However, there is currently no clear delineation of the structural changes that underpin this functional separation, as a DmsABC structure is not available for either enzyme type, and it is likely that a combination of altered regulatory patterns (substrate- versus ROS/RCS-based induction) and adjustments of substrate preferences accounts for the functional changes of DmsABC in bacterial pathogens.

Adaptations leading to altered substrate specificities are essential as they enable enzymes to access new cellular roles, but may be hard to predict as they can involve very few amino acid changes and occur in the substrate-binding regions of the enzymes rather than at the catalytic site, as proposed for *H. influenzae* MtsZ [55]. Such subtle changes are difficult to identify by sequence analysis, and to link to specific functions without biochemical studies and could lead to enzyme diversity being underestimated during sequence analyses.

Another interesting aspect of the virulence-associated S-oxide reductases is their ability to also convert biologically relevant N-oxides with high specificity, suggesting that these reactions may be as important to cellular survival as their ability to convert S-oxides [33,45]. It would be interesting to determine whether Dor- and Dms-type DMSO reductases from environmental bacteria can also convert these N-oxide substrates with high specificity, or whether this is a host-interaction-specific adaptation.

However, not all open question concern the Mo-containing S-oxide reductases: The cellular location of Msr-type enzymes and how the different localisations alter enzyme function and support bacterial survival in specific host niches has not been fully elucidated. This is important given the generally cytoplasmic location of MsrA and MsrB enzymes, cell envelope location of MsrAB fusion proteins, and the negligible contribution to stress resistance of the functionally related MsrP to virulence in *Salmonella* [4,41]. An improved understanding of the interplay between enzyme location and cellular function, as well as the protein substrates these enzymes repair, would support the exploitation of the potential of S-/N-oxide reductases as drug targets. The *Neisseria* MsrAB protein has already been proposed as a vaccine candidate [9], and in addition, the Mo-containing S-oxide reductases could be attractive drug targets as they have no homologues in human cells and play a significant role in host survival of pathogens.

## Perspectives

- Importance to the field: S-oxide reductases are emerging as essential determinants of bacterial in-host survival but remain linked to bacterial energy and redox metabolism. These close connections between virulence and cellular metabolic health are, as yet, understudied in many bacterial pathogens but represent essential functions that support infection persistence and are ‘switched on’ in response to exposure to the host environment.
- Summary: Bacterial S-oxide reductases belong to two types that catalyse S-oxide reduction via thiol- or Mo-chemistry. Virulence-associated S-oxide reductases are generally induced by oxidative stressors, such as hypochlorite, and often use quinols as electron donors, which makes them components of the bacterial respiratory chain. As a result, the enzymes serve a dual function during infection: They repair damage to proteins, nutrients, and precursors for biosynthesis, which is beneficial for bacterial cell integrity and nutrition, but their activity also provides bacteria with an additional route for redox balancing and respiration in the host environment, promoting optimal function of the bacterial metabolic network that is driven by the conditions encountered in the host.
- Future Directions: While it is well understood that enzymes that use quinols as electron donors or acceptors contribute to bacterial respiration, the follow-on benefits from these activities in the host environment are less well understood but can extend beyond the respiratory processes to enabling the use of substrates such as L-lactate that cannot be accessed without respiratory activity or another electron sink. There is also only a limited understanding of the functional changes that distinguish virulence-related S-oxide reductases from those that have purely metabolic roles. Future work should focus on understanding the wider cellular implications of S-oxide reductase activity in bacterial pathogens, as well as the significance of different enzyme structures and functions. This will not only provide biological insights but also enable the exploitation of these enzyme groups as potential drug targets.

## Open Access

Open access for this article was enabled by the participation of University of Queensland in an all-inclusive Read & Publish agreement with Portland Press and the Biochemical Society under an agreement with CAUL.

## Abbreviations

DMSO: Dimethylsulfoxide
RCS: reactive chorine species
ROS: reactive oxygen species
TMAO: Trimethylamine-N-oxide
